# An analysis of energy efficiency of the Pearl River Delta of China based on super-efficiency SBM model and Malmquist index

**DOI:** 10.1007/s11356-022-23465-z

**Published:** 2022-10-12

**Authors:** Boyu Liu

**Affiliations:** School of Social Sciences, The University of Manchester, Manchester, M13 9PL UK

**Keywords:** Energy efficiency, Super-efficiency SBM model, Malmquist index, Pearl River Delta

## Abstract

With the rapid growth rate of China’s economy, the extensive pattern of economic growth of “high energy consumption and low output” has magnified the constraints of energy issues on China’s economic development and environmental protection, which highlights the importance of improving energy efficiency. As one of the three major economic zones in China, the Pearl River Delta region also faces high energy consumption and pollution emissions while developing at a high speed. Hence, improving the energy efficiency of the Pearl River Delta region is needed, as it not only is conducive to driving its development of the surrounding green economy, but also promotes the subsequent sustainable economic development. However, there are few literatures on the calculation and analysis of energy efficiency in the Pearl River Delta, and lacking a systematic analysis of input–output index system of energy efficiency measurement. Therefore, this paper calculates the energy efficiencies and the Malmquist indexes based on the panel data of the nine cities in the Pearl River Delta from 2005 to 2019 through super-efficiency SBM model and Malmquist index method by using MAXDEA and MATLAB software. The result illustrates that all regions in the Pearl River Delta except Guangzhou and Shenzhen show obvious energy inefficiency, which is mainly caused by the imbalance between technical efficiency and scale efficiency. Based on the calculation results, this paper gives some relevant suggestions for the further approach of energy reform in the Pearl River Delta according to the calculation results.

## Introduction

Since China’s reform and opening up in 1978, China’s GDP grew at an average annual rate of 13.34%, showing rapid economic development growth rate. However, with the fast development of China’s economy, the negative impact of energy problem on China’s economic development and the protection of environment is becoming more and more obvious. On the one hand, the production mode of high energy input and low output has caused a lot of energy waste, which influence China’s sustainable economic development. On the other hand, over reliance on non-renewable energy and over consumption of energy resources have high negative impact on China’s ecological environment deterioration (Wei and Shen 2007). Under the circumstances of global energy shortage and imminent environmental problems, the extensive pattern of economic growth has no development advantages in the follow-up development, and the economic activities with low energy efficiency will have high negative externalities on the environment, people’s daily life, and further economic development. Fatih Birol, director of International Energy Agency (IEA), also proposed that energy efficiency is the core of coping with today’s global energy crisis, which can make energy supply more affordable, safer, and sustainable. Hence, how to improve the supply side structure, the energy efficiency, and the efficiency of economic growth and solve the environmental pollution correctly and efficiently are important questions for China to overcome, so as to achieve the goal of sustainable development of resources and economy. It is also an indispensable approach of China’s transformation process from high-speed economic growth to high-quality economic growth (Wang et al. 2010). In addition, due to the large regional gap in China and the huge differences in energy structure and industrial structure in different regions, it is essential to analyze the energy efficiency of different regions according to their economic and regional characteristics. It is also important to establish the reasons for their low or high energy efficiency, and point out the further development policy according to their characteristics.

As one of the three major urban agglomerations in China, the Pearl River Delta is the core area of China’s “the Belt and Road” economic development strategy and the construction of Guangdong-Hong Kong-Macao Great Bay area, with fast growth rate of economy. In 2020, the total GDP of the Pearl River Delta region was 8952.39 billion yuans, accounting for 8.83% of China’s total GDP. However, due to the agglomeration of industries in the Pearl River Delta, energy consumption and pollutant emissions are also extensive. To deal with the environmental problems, the outline of the reform and development plan for the Pearl River Delta region (2008–2020) launched by the Guangdong provincial government also pointed out that Guangdong shall strengthen resource conservation and environmental protection by vigorously developing the circular economy, strengthening pollution prevention and control. Therefore, in-depth research on the energy efficiency of the Pearl River Delta is of great significance to promote the development of new energy economy in the Pearl River Delta and assist the construction of regional ecological civilization. It can also drive the coordinated development of surrounding areas and give impetus to the construction of Guangdong-Hong Kong-Macao Great Bay area and “the Belt and Road.”

There are many methods for measuring energy efficiency, and some of them judge the extent to which the actual output capacity can reach the maximum output under the current fixed energy input, or the extent to which the minimum input can be achieved under the fixed output condition by determining the production function. However, the properties of the production frontier curve required to measure energy efficiency are often unknown, and real data are needed for subjective judgment and analysis, hence the results will be affected by the errors generated by subjective judgment and fitting the production function. In view of the above problems, the data envelopment analysis (DEA) analysis method proposed by Charnes and Cooper (1978) can be effectively avoided. DEA analysis method is established to judge whether the state is effective by considering a variety of resource inputs and actual outputs to build a non-parametric envelope along the front line, which is generally used to measure the production efficiency of some decision-making departments. As it does not need to consider the specific form of the model and the characteristics of relevant parameters, it effectively avoids the subjective impact of artificially determining the weight on the measurement results. Hence, it has been widely used in the evaluation of relevant policies and technical efficiency. Many scholars calculated and analyzed China’s overall energy consumption through DEA model and its derivatives combined with Malmquist index method, time series or spatial econometric model (Tu and Liu 2011; Zhang et al. 2004). However, DEA model cannot explain the impact of unexpected output on energy efficiency like slack-based model (SBM), and further analyze the impact of technological progress and scale effect on energy efficiency like Malmquist index (MI) method. Nevertheless, few scholars combined super-efficiency SBM model with MI method to calculate the energy efficiency. Meanwhile, most scholars mainly regard capital stock, labor and energy consumption as inputs to economic production, in which energy consumption generally only includes the consumption of fossil energy (Ke et al. 2011; Liu and Li 2009; Ma et al. 2011). They did not consider the input of bio-energy such as crop straw. In order to make the calculation results accurate and close to the reality, this paper combines the super-efficiency SBM model and MI method, and introduces the unexpected output and bio-energy input to calculate. The calculation results will be estimated by the MAXDEA and MATLAB software; then, this paper will give suggestions for improvement of the Pearl River Delta based on the calculation results and analysis.

This paper mainly includes six parts. In the second part, the literature review will be summarized. In third part, this paper will illustrate the super-efficiency SBM model and the Malmquist index theory, and list the indexes selection method as well as the data sources. In the fourth part, the calculation results through MAXDEA software will be shown. The results will be discussed in the fifth part, and conclusion as well as further research will be mentioned in the sixth and seventh part.

## Literature review

To solve the problems of resource shortage and serious environmental pollution, many scholars have emphasized the importance of energy efficiency and discussed how to improve energy efficiency from different perspectives. Subsequently, how to quantify energy efficiency begins to be one of the major issues in the academic community. Hence, some scholars have analyzed how to calculate the energy efficiency and how to improve the energy efficiency of various regions by analyzing the correlation between various factors.

Since the oil crisis in the 1970s, energy has gradually become a global issue of common concern. IEA pointed out that the current social energy shortage, energy security, energy prices and other challenges are intertwined with environmental pollution and climate crisis, and improving energy efficiency is more urgent than ever before. To illustrate the importance of energy efficiency, Tajudeen et al. (2018) proposed a two-step method to investigate the impact of energy efficiency improvement on carbon dioxide emissions, so as to explain the effect of energy efficiency on the environment. They used STSM and LSDVC models to examine and quantify the impact of energy efficiency on carbon dioxide emissions. The results show that improving energy efficiency contributes the most to reducing carbon dioxide emissions, indicating the essential role of energy efficiency in reducing emissions and improving environmental situation. Ahmadi et al. (2022a, b) also made a comprehensive discussion on the energy structure, existing installed capacity of renewable energy and related policies of Asian countries, and pointed out that most Asian countries have incalculable potential of renewable energy. They also pointed out that the field of renewable energy has great prospects for development, as it can attract investment, encourage technology and improve the environment. Meanwhile, Ahmadi et al. (2022a, b) further explored and optimized the application of artificial intelligence in energy system computing. Regarding the energy problem, Zahedi et al. (2022) believed that because hydroelectric power plants, as a kind of sustainable energy, have the advantage of producing less pollutants, decision-makers can increase energy output and energy efficiency by improving the efficiency of hydroelectric power generation. The slight negative environmental impact, they suggest, can be reduced by microalgae. At the same time, Daneshgara and Zahedi (2022) developed a dynamic production profitability model to model the operation of hydropower reservoir systems and the process of producer profitability in order to reduce the contribution of hydropower plant operators to maximize profits in the electricity market and improve local energy use efficiency. Saha et al. (2022) cut into the energy efficiency problem from the perspective of decarbonization of logistics distribution, and proposed a new multi-criteria group decision making method with double hesitant fuzzy (DHF) set to solve the problem that sustainable urban logistics prioritized zero-emission last-mile delivery (LMD) solution. Attari et al. (2022) discussed energy efficiency from the perspective of power supply chain, used grounded theory (GT) method to review and classify the literature, and pointed out the direction for future research on improving energy efficiency in the field of supply chain power.

As for the problem of how to effectively and accurately measure energy efficiency, scholars have different views. Patterson (1996) proposed that energy efficiency refers to the production of the same number of services or useful outputs with less energy, which can be measured by various quantitative indicators. Patterson (1996) also classified energy productivity according to different variables according to the production process from input to output. Most of the subsequent scholars analyzed the traditional indicators of energy physical input and GDP output system. In the case of determining the input–output indicators, the production frontier can be constructed by combining the actual quantity of input–output with the economic growth model, as it measures the optimal production possible boundary that can be achieved without the loss of energy efficiency. On this basis, researchers can calculate energy productivity through single factor and multi factor calculation methods, or calculate energy efficiency through input method and output method. However, the above measurement methods of energy efficiency require scholars to master the properties and parameter sizes of the unknown production front curve at first, which are affected by subjective factors, resulting in the lack of contrast of errors. In order to eliminate the influence of parameter settings on the calculation results, Farrell (1957) proposed that the energy efficiency or the efficiency of an industry and technology can be estimated by constructing a non-parametric segment convex surface, that is, data envelopment analysis (DEA). As the research method of DEA and its extended model effectively avoids the subjective setting of weights, simplifies the calculation process and reduces the calculation error, it is widely used in the evaluation technology and industrial efficiency (Wei and Shen 2007). Among them, Tone (2001) introduced unexpected output and slack variables into the traditional DEA model, and the improved super-efficiency slack-based model (SBM) model is often used to measure energy efficiency or ecological efficiency since it can take the negative externalities of economic production on the environment into account and is not affected by slack issues. For example, Gong et al. (2015), Wang and Wu (2011), Pan and Ying (2013) , and Yang et al. (2014) respectively used the super-efficiency SBM model to calculate China’s provincial industrial fossil energy efficiency, the temporal and spatial differences of China’s provincial ecological efficiency, China’s agricultural ecological efficiency, and China’s urban land using efficiency in the Yangtze River Delta region. However, some scholars mentioned that it is difficult to explain the economic significance of energy efficiency and its relationship with other indicators simply by measuring energy efficiency (Tone and Tsutsui 2010). Therefore, some scholars combined Malmquist index proposed by Malmquist (1953) and energy efficiency calculated by DEA model to explore the dynamic relationship among total factor energy efficiency (TFP), structural efficiency, energy and ecological efficiency. Yan et al. (2022) studied the energy efficiency in the Belt and Road region of China by combining the super-efficiency SBM model with the Malmquist index method. The results show that there are significant differences in energy efficiency among provinces along the Belt and Road in China, and it is greatly affected by technological progress. Meanwhile, other scholars also studied the static, temporal and spatial distribution characteristics of energy or ecological efficiency through Tobit regression model, time series, spatial econometric model and other methods (Zhang et al. 2004; Wei and Shen 2007; Ye et al. 2017).

On the other hand, the measurement and definition of energy input and output is also crucial. Since there is no unified standard for the concept and connotation of energy efficiency, coupled with the statistical differences of various input–output data, the results measured by different definitions of energy efficiency are often quite different. Hence, it is significant to determine the input–output indicators. As for the output indicators, although many scholars have different views on the system of taking GDP as energy output, most studies still take GDP as an economic output indicator to measure regional energy consumption for economic production (Wei and Shen 2007). Some scholars added other indicators besides GDP, such as regional fiscal revenue or employment rate and other social welfare as one of the output indicators (Ma et al. 2018). As for the unexpected output, some scholars used carbon emissions as the unexpected output of energy consumption (Wei and Shen 2007). However, other scholars pointed out that in the process of energy consumption, in addition to carbon emissions, there are other pollutants, such as sewage emissions, sulfur dioxide emissions, dust emissions and solid pollutant emissions. All of those have bad impact on the environment, and should also be included (Wang and Wu 2011). As for the input indicators, according to the basic theory of economic growth model, capital, labor and other production factors are indispensable production factors in the process of economic production (Ke et al. 2011; Liu and Li 2009; Ma et al. 2011). In addition to the investment of capital stock, labor force, and energy consumption that most scholars have considered, many scholars also took urban water resources investment, urban construction area and knowledge stock as input indicators to measure energy efficiency (Yang et al. 2014; Gong et al. 2015). In addition, Hu and Wang (2006) proposed that generally calculated energy data only include traditional fossil energy, excluding low-carbon bio-energy, such as crop straw. Therefore, Shi and Shen (2008) mentioned that bio-energy should also be included in the energy input index for calculation. In conclusion, scholars were partly the same in the establishment of input–output indicators. Most scholars regarded GDP as the output of economic production, pollutants including water pollution and exhaust gas pollution as unexpected output indicators, and capital stock, labor and energy consumption as input indicators of economic production.

In conclusion, many scholars have conducted extensive research on the importance of energy efficiency to the environment and how to improve energy efficiency from different aspects in the research of energy efficiency. However, there are still many problems in the measurement of energy efficiency. First, many scholars assume the production function through existing data to calculate energy efficiency, which will be affected by subjective factors. Secondly, most schools do not consider the unexpected output of economic production such as pollution emissions and the energy consumption of non-fossil energy such as bio energy. Finally, many scholars only calculate the energy efficiency of each country or region as a whole, and there is no relevant research in the Pearl River Delta. The existing research lacks targeted measurement, and the conclusions and suggestions given through overall measurement may not be applicable to relevant regions, but may further expand the gap. In order to calculate the energy efficiency of the Pearl River Delta more accurately, this paper introduces unexpected outputs and bio-energy inputs on the basis of the previous literature. Meanwhile, the value of energy efficiencies and Malmquist indexes of the nine cities in the Pearl River Delta from 2005 to 2019 is calculated by combining the super-efficiency SBM model with Malmquist index method through MAXDEA and MATLAB software. According to the calculation results, this paper analyzes the dynamic trend of energy efficiency in the Pearl River Delta and gives relevant suggestions for future development.

## Method and data

### Model

#### Super-efficiency SBM model

The traditional DEA analysis method proposed by Charnes and Cooper (1978) assumed that there is a production technology with constant return to scale to study the technical efficiency of *k* decision-making units (DMU) when they input *m* factors to produce *n* outputs. Each decision-making unit *i* (*i* = 1, 2, …, *k*) corresponds to an input vector ${\mathrm{X}}_{\mathrm{i}}={({\mathrm{x}}_{1\mathrm{i}}, {\mathrm{x}}_{2\mathrm{i}}, \dots , {\mathrm{x}}_{\mathrm{mi}})}^{\mathrm{T}}$ and an output vector ${\mathrm{Y}}_{\mathrm{i}}={({\mathrm{y}}_{1\mathrm{i}}, {\mathrm{y}}_{2\mathrm{i}}, \dots , {\mathrm{y}}_{\mathrm{ni}})}^{\mathrm{T}}$. ${\mathrm{x}}_{\mathrm{si}}$ is the total input of the *i*th DMU to the *s*th type input, where ${\mathrm{x}}_{\mathrm{si}}>0$ for all *s* (s = 1, 2, …, *m*), and ${\mathrm{y}}_{\mathrm{ri}}$ is the total output of the *i*th DMU to the *r*th type output, where ${\mathrm{y}}_{\mathrm{ri}}>0$ for all *r* (*r* = 1, 2, …, *n*). In order to maximize production efficiency and reduce unnecessary losses, ${\mathrm{i}}_{0}$ th DMU needs to solve the following energy planning problems:

$$\mathrm{Min \theta }$$

Subject to the budget constraints:

$${\sum }_{\mathrm{i}=1}^{\mathrm{k}}{\mathrm{X}}_{\mathrm{i}}{\upgamma }_{\mathrm{i}}\le\uptheta {\mathrm{X}}_{{\mathrm{i}}_{0}}$$

$${\sum }_{\mathrm{i}=1}^{\mathrm{k}}{\mathrm{Y}}_{\mathrm{i}}{\upgamma }_{\mathrm{i}}\ge {\mathrm{Y}}_{{\mathrm{i}}_{0}}$$

$${\upgamma }_{\mathrm{i}}\ge 0,\mathrm{ \theta is free},\mathrm{ for i}=1, 2, \dots ,\mathrm{ k}$$

${\mathrm{X}}_{{\mathrm{i}}_{0}}$ and ${\mathrm{Y}}_{{\mathrm{i}}_{0}}$ represent the input and output vector of ${\mathrm{i}}_{0}$ th DMU; $\theta$ represents the investment reduction ratio; $\gamma$ represents the linear combination of decision-making units. The solved value of $\theta$ is called the efficiency value of ${\mathrm{i}}_{0}$ th DMU, which is between 0 and 1. The unit is technically effective only when $\theta =1$.

However, the traditional radial and angular DEA model did not take the slack issues of input–output indicators and the negative external effects of the environment in the production process into account, which will lead to overestimation or excessive error of the prediction results. In order to solve the problem of input–output variable slack, Tone (2001) added slack variables to the traditional DEA model and proposed a non-radial, non-angular slack-based model (SBM). In this paper, the SBM model is introduced as the basic model for measuring energy efficiency. To illustrate the negative environmental external effects produced by the production process, g negative outputs in the production process are added into the model as unexpected outputs. Each decision-making unit *i* corresponds to an unexpected output vector ${\mathrm{U}}_{\mathrm{i}}={({\mathrm{u}}_{1\mathrm{i}}, {\mathrm{u}}_{2\mathrm{i}}, \dots , {\mathrm{u}}_{\mathrm{gi}})}^{\mathrm{T}}$, where ${\mathrm{u}}_{\mathrm{li}}$ is the total unexpected output of the *i*th DMU to the *l*th type unexpected output, and ${\mathrm{u}}_{\mathrm{li}}>0$ for all *l* (*l* = 1, 2, …, *g*). Then, the energy planning problems for ${\mathrm{i}}_{0}$ th DMU is:

1 $$\mathrm{Min \rho }=\frac{1-\frac{1}{\mathrm{m}}{\sum }_{\mathrm{s}=1}^{\mathrm{m}}\frac{{\mathrm{s}}_{\mathrm{s}}^{\mathrm{x}}}{{\mathrm{x}}_{\mathrm{si}}}}{1+\frac{1}{\mathrm{n}+\mathrm{g}}({\sum }_{\mathrm{r}=1}^{\mathrm{n}}\frac{{\mathrm{s}}_{\mathrm{r}}^{\mathrm{y}}}{{\mathrm{y}}_{\mathrm{ri}}}+{\sum }_{\mathrm{l}=1}^{\mathrm{g}}\frac{{\mathrm{s}}_{\mathrm{l}}^{\mathrm{u}}}{{\mathrm{u}}_{\mathrm{li}}})}$$

Subject to the budget constraints:

$$\begin{array}{c}{\mathrm{x}}_{{\mathrm{si}}_{0}}={\sum }_{\mathrm{i}=1}^{\mathrm{k}}{\mathrm{x}}_{\mathrm{si}}{\upgamma }_{\mathrm{i}}+{\mathrm{s}}_{\mathrm{s}}^{\mathrm{x}}\\ {\mathrm{y}}_{{\mathrm{ri}}_{0}}={\sum }_{\mathrm{i}=1}^{\mathrm{k}}{\mathrm{y}}_{\mathrm{ri}}{\upgamma }_{\mathrm{i}}-{\mathrm{s}}_{\mathrm{r}}^{\mathrm{y}}\\ {\mathrm{u}}_{{\mathrm{li}}_{0}}={\sum }_{\mathrm{i}=1}^{\mathrm{k}}{\mathrm{u}}_{\mathrm{li}}{\upgamma }_{\mathrm{i}}+{\mathrm{s}}_{\mathrm{l}}^{\mathrm{u}}\end{array}$$

$${\upgamma }_{\mathrm{i}}, {\mathrm{s}}_{\mathrm{s}}^{\mathrm{x}}, {\mathrm{s}}_{\mathrm{r}}^{\mathrm{y}}, {\mathrm{s}}_{\mathrm{l}}^{\mathrm{u}}\ge 0,\mathrm{ for i}=1, 2, \dots ,\mathrm{ k},\mathrm{ s}=1, 2, \dots ,\mathrm{ m},\mathrm{ r}=1, 2, \dots ,\mathrm{ n},\mathrm{ l}=1, 2, \dots ,\mathrm{ g}$$

${\mathrm{s}}_{\mathrm{s}}^{\mathrm{x}}$, ${\mathrm{s}}_{\mathrm{r}}^{\mathrm{y}}$, and ${\mathrm{s}}_{\mathrm{l}}^{\mathrm{u}}$ represent the input redundancy, positive output insufficiency, and unexpected output superscalar of ${\mathrm{i}}_{0}$ th DMU. The solved value of $\rho$ represents the efficiency value of ${\mathrm{i}}_{0}$ th DMU, which is between 0 and 1. The unit is technically effective in SBM only when $\rho =1$, which means that ${\mathrm{s}}_{\mathrm{s}}^{\mathrm{x}}, {\mathrm{s}}_{\mathrm{r}}^{\mathrm{y}}, {\mathrm{s}}_{\mathrm{l}}^{\mathrm{u}}=0$. However, this method did not consider the comparison between efficient DMUs. Hence, this paper introduces the method of super-efficiency SBM to solve the problem.

When discussing the super-efficiency SBM, this paper supposes that ${\mathrm{i}}_{0}$ th DMU is effective in SBM. Then, the super-efficient SBM model with unexpected output can be expressed as follows:

2 $$\mathrm{Min \varphi }=\frac{\frac{1}{\mathrm{m}}{\sum }_{\mathrm{s}=1}^{\mathrm{m}}\frac{\overline{\mathrm{x}}}{{\mathrm{x}}_{\mathrm{si}}}}{\frac{1}{\mathrm{n}+\mathrm{g}}({\sum }_{\mathrm{r}=1}^{\mathrm{n}}\frac{\overline{\mathrm{y}}}{{\mathrm{y}}_{\mathrm{ri}}}+{\sum }_{\mathrm{l}=1}^{\mathrm{g}}\frac{\overline{\mathrm{u}}}{{\mathrm{u}}_{\mathrm{li}}})}$$

Subject to the budget constraints:

$$\begin{array}{c}\overline{\mathrm{x}}\ge {\sum }_{\mathrm{i}=1,\mathrm{ i}\ne {\mathrm{i}}_{0}}^{\mathrm{k}}{\mathrm{x}}_{\mathrm{si}}{\upgamma }_{\mathrm{i}}\\ \overline{\mathrm{y}}\le {\sum }_{\mathrm{i}=1,\mathrm{ i}\ne {\mathrm{i}}_{0}}^{\mathrm{k}}{\mathrm{y}}_{\mathrm{ri}}{\upgamma }_{\mathrm{i}}\\ \begin{array}{c}\overline{\mathrm{u}}\ge {\sum }_{\mathrm{i}=1,\mathrm{ i}\ne {\mathrm{i}}_{0}}^{\mathrm{k}}{\mathrm{u}}_{\mathrm{li}}{\upgamma }_{\mathrm{i}}\\ {\upgamma }_{\mathrm{i}}\ge 0,\;\mathrm{ for\;i}=1, 2, \dots ,\;\mathrm{ k}\\ \begin{array}{c}\overline{\mathrm{x}}\ge {\mathrm{x}}_{{\mathrm{si}}_{0}},\;\mathrm{ s}=1, 2, \dots ,\;\mathrm{ m}\\ \overline{\mathrm{y}}\le {\mathrm{y}}_{{\mathrm{ri}}_{0}},\;\mathrm{ r}=1, 2, \dots ,\;\mathrm{ n}\\ \overline{\mathrm{u}}\ge {\mathrm{u}}_{{\mathrm{li}}_{0}},\;\mathrm{ l}=1, 2, \dots ,\;\mathrm{ g}\end{array}\end{array}\end{array}$$

This paper use models (1) and (2) to calculate the energy efficiency (EE) in the Pearl River Delta from 2005 to 2019. The energy efficiency of region *i* at time *t* can be expressed as:

$$\begin{array}{c}{\mathrm{EE}}_{\mathrm{jt}}=\left\{\begin{array}{c}{\uprho }_{\mathrm{jt}}, \;when \;{\uprho }_{\mathrm{jt}}<1\\ {\mathrm{\varphi }}_{\mathrm{jt}}, \;when\;{\uprho }_{\mathrm{jt}}=1\end{array}\right.\\ j=1, 2, \dots , 9, t=2005, 2006, \dots , 2019\end{array}$$

Compared with other calculation methods, the super-efficient SBM model can not only eliminate the influence of subjective factors and external conditions on the calculation results, but also effectively solve the result deviation caused by relaxation variables. Meanwhile, compared with the traditional SBM model, the super-efficient SBM model can compare the completely efficient decision-making units, which increases the interpretability and persuasion of the results. Besides, compared with the efficiency evaluation of the general standard model, this paper adds unexpected outputs such as water pollution emissions, and further studies the input indicators, making the calculation results closer to reality.

#### Malmquist index theory

Malmquist index method is proposed on the basis of DEA method, and uses the ratio of distance function to calculate input–output efficiency. As it can not only investigate the change of regional production technology progress (TC), but also further decompose the change of technical efficiency (TEC) into pure technical efficiency change (PEC) and scale efficiency change (SEC), so as to better understand the composition of productivity and its dynamic change trend, it is often used to analyze changes in energy efficiency in various regions. The relationship of input and output is expressed as $({\mathrm{X}}^{\mathrm{t}}, {\mathrm{Y}}^{\mathrm{t}})$, and comprehensive efficiency level of the frontier of *i*th DMU in t period when taking the technology in t period as a reference is expressed as ${\mathrm{D}}_{\mathrm{i}}^{\mathrm{t}}({\mathrm{x}}^{\mathrm{t}}, {\mathrm{y}}^{\mathrm{t}})$. According to Fare and Grosskopf (1992) and Fare et al. (1994b, a), the Malmquist index from period *t* to period *t* + 1 based on the reference technique of period *t* and period *t* + 1, which indicates the change in the overall efficiency level, is the ratio of the comprehensive efficiency levels in *t* period and *t* + 1 period:

$$\begin{array}{c}{\mathrm{M}}_{\mathrm{t}}({\mathrm{x}}^{\mathrm{t}}, {\mathrm{y}}^{\mathrm{t}}, {\mathrm{x}}^{\mathrm{t}+1}, {\mathrm{y}}^{\mathrm{t}+1})=\frac{{\mathrm{D}}^{\mathrm{t}}({\mathrm{x}}^{\mathrm{t}+1}, {\mathrm{y}}^{\mathrm{t}+1})}{{\mathrm{D}}^{\mathrm{t}}({\mathrm{x}}^{\mathrm{t}}, {\mathrm{y}}^{\mathrm{t}})}\\ {\mathrm{M}}_{\mathrm{t}+1}({\mathrm{x}}^{\mathrm{t}}, {\mathrm{y}}^{\mathrm{t}}, {\mathrm{x}}^{\mathrm{t}+1}, {\mathrm{y}}^{\mathrm{t}+1})=\frac{{\mathrm{D}}^{\mathrm{t}+1}({\mathrm{x}}^{\mathrm{t}+1}, {\mathrm{y}}^{\mathrm{t}+1})}{{\mathrm{D}}^{\mathrm{t}+1}({\mathrm{x}}^{\mathrm{t}}, {\mathrm{y}}^{\mathrm{t}})}\end{array}$$

Caves et al. (1982) pointed out that with the symmetrical economic meanings of the above two Malmquist indexes, according to the ideal index idea, the comprehensive productivity index can be obtained by calculating their geometric average:

$$\begin{array}{c}\mathrm{M}({\mathrm{x}}^{\mathrm{t}}, {\mathrm{y}}^{\mathrm{t}}, {\mathrm{x}}^{\mathrm{t}+1}, {\mathrm{y}}^{\mathrm{t}+1})={({\mathrm{M}}_{\mathrm{t}}({\mathrm{x}}^{\mathrm{t}}, {\mathrm{y}}^{\mathrm{t}}, {\mathrm{x}}^{\mathrm{t}+1}, {\mathrm{y}}^{\mathrm{t}+1})\bullet {\mathrm{M}}_{\mathrm{t}+1}({\mathrm{x}}^{\mathrm{t}}, {\mathrm{y}}^{\mathrm{t}}, {\mathrm{x}}^{\mathrm{t}+1}, {\mathrm{y}}^{\mathrm{t}+1}))}^\frac{1}{2}\\ {=(\frac{{\mathrm{D}}^{\mathrm{t}}({\mathrm{x}}^{\mathrm{t}+1}, {\mathrm{y}}^{\mathrm{t}+1})}{{\mathrm{D}}^{\mathrm{t}}({\mathrm{x}}^{\mathrm{t}}, {\mathrm{y}}^{\mathrm{t}})}\bullet \frac{{\mathrm{D}}^{\mathrm{t}+1}({\mathrm{x}}^{\mathrm{t}+1}, {\mathrm{y}}^{\mathrm{t}+1})}{{\mathrm{D}}^{\mathrm{t}+1}({\mathrm{x}}^{\mathrm{t}}, {\mathrm{y}}^{\mathrm{t}})})}^\frac{1}{2}\end{array}$$

The Malmquist index indicates *i*th DMU’s change degree of productivity from period *t* to period *t* + 1. The productivity shows an upward trend when $M>1$, and shows a downward trend when $M<1$. According to Fare et al. (1994b, a), the Malmquist index can be decomposed into comprehensive technical efficiency change index (TEC) and technical progress index (TC) under the assumption of constant return to scale:

$$\begin{array}{c}{\mathrm{M}}_{\mathrm{c}}({\mathrm{x}}^{\mathrm{t}}, {\mathrm{y}}^{\mathrm{t}}, {\mathrm{x}}^{\mathrm{t}+1}, {\mathrm{y}}^{\mathrm{t}+1})={(\frac{{\mathrm{D}}_{\mathrm{c}}^{\mathrm{t}}({\mathrm{x}}^{\mathrm{t}+1}, {\mathrm{y}}^{\mathrm{t}+1})}{{\mathrm{D}}_{\mathrm{c}}^{\mathrm{t}}({\mathrm{x}}^{\mathrm{t}}, {\mathrm{y}}^{\mathrm{t}})}\bullet \frac{{\mathrm{D}}_{\mathrm{c}}^{\mathrm{t}+1}({\mathrm{x}}^{\mathrm{t}+1}, {\mathrm{y}}^{\mathrm{t}+1})}{{\mathrm{D}}_{\mathrm{c}}^{\mathrm{t}+1}({\mathrm{x}}^{\mathrm{t}}, {\mathrm{y}}^{\mathrm{t}})})}^\frac{1}{2}\\ =\frac{{\mathrm{D}}_{\mathrm{c}}^{\mathrm{t}+1}({\mathrm{x}}^{\mathrm{t}+1}, {\mathrm{y}}^{\mathrm{t}+1})}{{\mathrm{D}}_{\mathrm{c}}^{\mathrm{t}}({\mathrm{x}}^{\mathrm{t}}, {\mathrm{y}}^{\mathrm{t}})}\bullet {(\frac{{\mathrm{D}}_{\mathrm{c}}^{\mathrm{t}}({\mathrm{x}}^{\mathrm{t}+1}, {\mathrm{y}}^{\mathrm{t}+1})}{{\mathrm{D}}_{\mathrm{c}}^{\mathrm{t}+1}({\mathrm{x}}^{\mathrm{t}+1}, {\mathrm{y}}^{\mathrm{t}+1})}\bullet \frac{{\mathrm{D}}_{\mathrm{c}}^{\mathrm{t}}({\mathrm{x}}^{\mathrm{t}}, {\mathrm{y}}^{\mathrm{t}})}{{\mathrm{D}}_{\mathrm{c}}^{\mathrm{t}+1}({\mathrm{x}}^{\mathrm{t}}, {\mathrm{y}}^{\mathrm{t}})})}^\frac{1}{2}=\mathrm{TEC}\bullet \mathrm{TC}\\ \begin{array}{c}\mathrm{TEC}=\frac{{\mathrm{D}}_{\mathrm{c}}^{\mathrm{t}+1}({\mathrm{x}}^{\mathrm{t}+1}, {\mathrm{y}}^{\mathrm{t}+1})}{{\mathrm{D}}_{\mathrm{c}}^{\mathrm{t}}({\mathrm{x}}^{\mathrm{t}}, {\mathrm{y}}^{\mathrm{t}})}\\ \mathrm{TC}={(\frac{{\mathrm{D}}_{\mathrm{c}}^{\mathrm{t}}({\mathrm{x}}^{\mathrm{t}+1}, {\mathrm{y}}^{\mathrm{t}+1})}{{\mathrm{D}}_{\mathrm{c}}^{\mathrm{t}+1}({\mathrm{x}}^{\mathrm{t}+1}, {\mathrm{y}}^{\mathrm{t}+1})}\bullet \frac{{\mathrm{D}}_{\mathrm{c}}^{\mathrm{t}}({\mathrm{x}}^{\mathrm{t}}, {\mathrm{y}}^{\mathrm{t}})}{{\mathrm{D}}_{\mathrm{c}}^{\mathrm{t}+1}({\mathrm{x}}^{\mathrm{t}}, {\mathrm{y}}^{\mathrm{t}})})}^\frac{1}{2}\end{array}\end{array}$$

${\mathrm{M}}_{\mathrm{c}}$ is calculated by supposing return to scale is constant (CRS), and ${\mathrm{M}}_{\mathrm{v}}$ is calculated by supposing return to scale is variable (VRS), so the ratio of the two evaluates the return to scale. Based on this, the technical efficiency change index (TEC) can be further decomposed into pure technical efficiency index (PEC) and scale efficiency index (SEC) (Fare et al. 1994b, a):

$$\begin{array}{c}{\mathrm{M}}_{\mathrm{v},\mathrm{c}}({\mathrm{x}}^{\mathrm{t}}, {\mathrm{y}}^{\mathrm{t}}, {\mathrm{x}}^{\mathrm{t}+1}, {\mathrm{y}}^{\mathrm{t}+1})=\frac{{\mathrm{D}}_{\mathrm{v}}^{\mathrm{t}+1}({\mathrm{x}}^{\mathrm{t}+1}, {\mathrm{y}}^{\mathrm{t}+1})}{{\mathrm{D}}_{\mathrm{v}}^{\mathrm{t}}({\mathrm{x}}^{\mathrm{t}}, {\mathrm{y}}^{\mathrm{t}})}\bullet (\frac{{\mathrm{D}}_{\mathrm{c}}^{\mathrm{t}+1}({\mathrm{x}}^{\mathrm{t}+1}, {\mathrm{y}}^{\mathrm{t}+1})}{{\mathrm{D}}_{\mathrm{c}}^{\mathrm{t}}({\mathrm{x}}^{\mathrm{t}}, {\mathrm{y}}^{\mathrm{t}})}\bullet \frac{{\mathrm{D}}_{\mathrm{v}}^{\mathrm{t}}({\mathrm{x}}^{\mathrm{t}}, {\mathrm{y}}^{\mathrm{t}})}{{\mathrm{D}}_{\mathrm{v}}^{\mathrm{t}+1}({\mathrm{x}}^{\mathrm{t}+1}, {\mathrm{y}}^{\mathrm{t}+1})})\\ \bullet {(\frac{{\mathrm{D}}_{\mathrm{c}}^{\mathrm{t}}({\mathrm{x}}^{\mathrm{t}+1}, {\mathrm{y}}^{\mathrm{t}+1})}{{\mathrm{D}}_{\mathrm{c}}^{\mathrm{t}+1}({\mathrm{x}}^{\mathrm{t}+1}, {\mathrm{y}}^{\mathrm{t}+1})}\bullet \frac{{\mathrm{D}}_{\mathrm{c}}^{\mathrm{t}}({\mathrm{x}}^{\mathrm{t}}, {\mathrm{y}}^{\mathrm{t}})}{{\mathrm{D}}_{\mathrm{c}}^{\mathrm{t}+1}({\mathrm{x}}^{\mathrm{t}}, {\mathrm{y}}^{\mathrm{t}})})}^\frac{1}{2}\\ \begin{array}{c}=\mathrm{TEC}\bullet \mathrm{TC}=\mathrm{PEC}\bullet \mathrm{SEC}\bullet \mathrm{TC}\\ \begin{array}{c}\mathrm{PEC}=\frac{{\mathrm{D}}_{\mathrm{v}}^{\mathrm{t}+1}({\mathrm{x}}^{\mathrm{t}+1}, {\mathrm{y}}^{\mathrm{t}+1})}{{\mathrm{D}}_{\mathrm{v}}^{\mathrm{t}}({\mathrm{x}}^{\mathrm{t}}, {\mathrm{y}}^{\mathrm{t}})}\\ \mathrm{SEC}=\frac{{\mathrm{D}}_{\mathrm{c}}^{\mathrm{t}+1}({\mathrm{x}}^{\mathrm{t}+1}, {\mathrm{y}}^{\mathrm{t}+1})}{{\mathrm{D}}_{\mathrm{c}}^{\mathrm{t}}({\mathrm{x}}^{\mathrm{t}}, {\mathrm{y}}^{\mathrm{t}})}\bullet \frac{{\mathrm{D}}_{\mathrm{v}}^{\mathrm{t}}({\mathrm{x}}^{\mathrm{t}}, {\mathrm{y}}^{\mathrm{t}})}{{\mathrm{D}}_{\mathrm{v}}^{\mathrm{t}+1}({\mathrm{x}}^{\mathrm{t}+1}, {\mathrm{y}}^{\mathrm{t}+1})}\end{array}\end{array}\end{array}$$

PEC, SEC, and TC indexes are called the decomposition indexes of the Malmquist index. If the calculated values of PEC, SEC, and TC are greater than 1, it indicates that their efficiencies from *t* to *t* + 1 have improved, and if they are less than 1, the result is just the opposite. When their values are equal to 1, it illustrates that pure technical efficiency, scale efficiency, and technical progress indexes remain the same.

By combining Malmquist index theory with the super-efficiency SBM method, this paper can calculate the value of Malmquist index representing the growth of energy efficiency based on the SBM method. Meanwhile, this paper can not only acquaint the energy condition of all regions, but also further analyze them through the perspectives of the change of pure technical efficiency, scale efficiency, and technical progress. Furthermore, due to the complexity of the calculation process, the results of the super-efficient SBM model and Malmquist index method are basically calculated by programs. At present, the widely used calculation program is MAXDEA software. This paper will use this software to calculate and test it through the DEA data package of MATLAB.

### Index selection and data source

#### Sample selection

According to the main cities defined in the outline of the reform and development plan of the Pearl River Delta (2008–2020), this paper selects Guangzhou, Shenzhen, Zhuhai, Foshan, Dongguan, Jiangmen, Zhaoqing, Zhongshan, and Huizhou as the decision-making units of the Pearl River Delta to analyze its energy efficiency. Due to the partial lack of municipal data, the maximum time span that can be obtained from the municipal data in the statistical yearbook of Guangdong Province and the statistical yearbook of each city is from 2005 to 2019. This paper selects the period from 2005 to 2019, and studies the regional differences, dynamic changes and sources of energy efficiency growth in the Pearl River Delta region of China through super-efficiency SBM model and Malmquist index method.

#### Input–output index selection and data source

The data in this paper are mainly collected from the statistical yearbooks of Guangdong Province and each city from 2005 to 2019. Some missing data are supplemented by Guotaian Database.

(A) Input indexes (X)

According to the neoclassical economic growth model, the main driving force of economic growth is capital stock, material and human capital input, so this paper takes the four indicators as input indicators. Since this paper studies energy efficiency, the material input index of this paper will be represented by the non-biological energy consumption and biological energy consumption of each region.

- Capital stock input
  To calculate the value of capital stock input, this paper introduces the basic estimation formula of productive capital stock, which can be expressed as ${\mathrm{K}}_{\mathrm{t}}={\mathrm{I}}_{\mathrm{t}}+(1-{\upsigma }_{\mathrm{t}}){\mathrm{K}}_{\mathrm{t}-1}$, where ${\mathrm{K}}_{\mathrm{t}}$ is the capital stock at time t, ${\mathrm{I}}_{\mathrm{t}}$ is the annual new fixed capital investment, and ${\upsigma }_{\mathrm{t}}$ is the capital depreciation rate at time t (Zhang et al. 2004). This paper assumes that the capital depreciation rate is fixed since 2005. As for the value of capital depreciation rate in China, there are two comparatively authoritative methods to calculate the fixed capital depreciation rate in China, which are the methods of Zhang et al. (2004) and Shan (2008), and the calculated fixed capital depreciation rate in China by the two methods are 9.6% and 10.96% with the same base year 1956. This paper has used these two methods to calculate, as the average value of the difference between the energy efficiency values calculated by the two methods is 0.0019, this paper only selects 9.6% as the capital depreciation rate in the Pearl River Delta region of China. As the earliest available data of fixed asset investment in various cities is in 1990, the base year of capital Stock calculation in this paper is 1990. The capital stock in the base year is calculated by dividing the fixed capital investment in that year by the sum of the growth rate and depreciation rate of capital investment (Zhang et al. 2004). The data of the annual new fixed capital investment is collected as the annual new fixed capital investment of each city from 1990 to 2019.
- Human capital input
  Human capital consists of labor force quantity and labor force education. Since the data of per capita education level in each city is not available, this paper uses the quantity of social employees of each region at the end of the year as its human capital at that time.
- Abiotic energy consumption
  Abiotic energy consumption includes the consumption of coal, oil, natural gas, hydropower and other energy. This paper uses the annual energy consumption recorded in the statistical yearbook of each city to express the energy input of each city.
- Bio-energy consumption

Hu and Wang (2006) proposed that since general energy data only include traditional disposable energy, such as coal, they do not cover low-carbon renewable energy, especially bio-energy. Due to the lack of coherent data statistics of bio-energy, this paper refers the method proposed by Hu and Wang (2006) that using the planting area of crops as the proxy variable of bio-energy. For the lack of data on the total sown area of crops in some cities, this paper uses the sown area of major crops in each city every year to measure their bio-energy consumption.

(B) Positive output index (Y)
  As for the input index, the main reason and final result of energy consumption is to promote regional economic development; hence, this paper takes the annual GDP of each city as the positive output of the city in that year.
(C) Unexpected output indexes (U)

In this paper, the environmental pollution caused by the production process is calculated as unexpected output. Three major pollution discharges, including sewage discharge, industrial waste gas discharge and solid waste discharge, in each city is taken as the direct embodiment of the negative externalities of the city’s energy consumption and production activities on the environment. Due to the lack of data on the production of industrial solid waste and industrial dust emissions in some cities in the Pearl River Delta, this paper mainly takes the annual sewage emissions and annual industrial exhaust emissions of each city as the unexpected output of the city in that year.

Referring to the literature review and the availability of data, the index system of energy efficiency constructed in this paper is shown in Table 1.

**Table 1:** Index system of energy efficiency

Index	Primary index	Unit of measurement	Symbol
Input variables	Employees of the whole society	10,000 people	${\mathrm{X}}_{1}$
	Annual capital stock	100 million RMB	${\mathrm{X}}_{2}$
	Annual energy consumption	10,000 tons of standard coal	${\mathrm{X}}_{3}$
	The sown area of major crops	Square kilometers	${\mathrm{X}}_{4}$
Positive output variables	Annual gross domestic product	100 million RMB	$\mathrm{Y}$
Unexpected output variables	Annual sewage emissions	Million tons	${\mathrm{U}}_{1}$
	Annual industrial exhaust emissions	100 million cubic meters	${\mathrm{U}}_{2}$

## Results

### Calculation results of super-efficiency SBM model

**Fig. 1:**
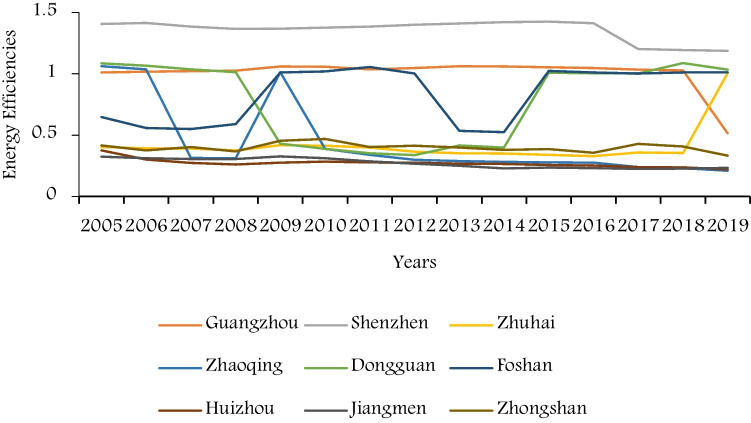
Energy efficiencies of Pearl River Delta cities (2005–2019)

In this paper, MAXDEA software is used to calculate the energy efficiency and Malmquist index, and the results are tested by the DEA data package of MATLAB. Through calculation, the calculation results of energy efficiencies of each city in the Pearl River Delta from 2005 to 2019 are shown in Table 2, and the trends of energy efficiencies of each city from 2005 to 2019 is shown in Fig. 1. Through the calculation results of MAXDEA software, the value of slack variables called the slack movement is summarized by the improvement direction of each slack variable, which shows increase and decrease improvement direction of slack variables for positive and negative value. The average value of slack movement in each region and each period for the 15 years are shown in Table 3. For inefficient DMUs, the slack variables of input and unexpected output are negative, indicating that the improvement direction is to reduce input, while the slack variables of output are positive, indicating that the improvement direction is to increase output. Note that in the super-efficiency SBM model, for the super-efficiency DMU (DMU with efficiency value greater than 1), the result of input slack variable is positive, while the result of output slack variable is negative. In order to effectively explain the energy efficiency of each region and time, this paper will analyze the energy situation of the whole Pearl River Delta region and the energy efficiency differences of each region combined with the following results.

**Table 2:** Energy efficiencies of Pearl River Delta cities (2005–2019)

Year	Guangzhou	Shenzhen	Zhuhai	Zhaoqing	Dongguan	Foshan	Huizhou	Jiangmen	Zhongshan	Average
2005	1.0111	1.4059	0.4013	1.0604	1.0847	0.6475	0.3753	0.3233	0.4148	0.7471
2006	1.0157	1.4133	0.3914	1.0351	1.0656	0.5571	0.3003	0.3108	0.3756	0.7183
2007	1.0215	1.3836	0.3901	0.3121	1.0350	0.5492	0.2726	0.3050	0.4033	0.6303
2008	1.0250	1.3652	0.3737	0.3101	1.0134	0.5904	0.2598	0.3044	0.3670	0.6232
2009	1.0591	1.3669	0.4163	1.0124	0.4301	1.0104	0.2747	0.3258	0.4528	0.7054
2010	1.0577	1.3752	0.4136	0.3883	0.3879	1.0195	0.2831	0.3108	0.4692	0.6339
2011	1.0352	1.3846	0.3982	0.3381	0.3523	1.0555	0.2762	0.2857	0.4028	0.6143
2012	1.0458	1.3994	0.3648	0.2978	0.3363	1.0023	0.2746	0.2657	0.4124	0.5999
2013	1.0616	1.4104	0.3506	0.2880	0.4148	0.5344	0.2654	0.2483	0.3985	0.5525
2014	1.0586	1.4213	0.3482	0.2809	0.3978	0.5243	0.2644	0.2282	0.3791	0.5447
2015	1.0532	1.4239	0.3390	0.2762	1.0081	1.0219	0.2560	0.2343	0.3861	0.6665
2016	1.0466	1.4125	0.3284	0.2739	1.0036	1.0111	0.2511	0.2311	0.3555	0.6571
2017	1.0333	1.2006	0.3573	0.2376	1.0025	1.0023	0.2380	0.2235	0.4268	0.6358
2018	1.0264	1.1937	0.3525	0.2307	1.0863	1.0102	0.2356	0.2267	0.4064	0.6410
2019	0.5147	1.1875	1.0040	0.2089	1.0342	1.0103	0.2186	0.2315	0.3332	0.6381
Average	1.0044	1.3563	0.4153	0.4367	0.7768	0.8364	0.2697	0.2703	0.3989	0.6405

**Table 3:** Average slack movement of each index of each region and each period

DMU	Score	${\mathrm{X}}_{1}$	${\mathrm{X}}_{2}$	${\mathrm{X}}_{3}$	${\mathrm{X}}_{4}$	$\mathrm{Y}$	${\mathrm{U}}_{1}$	${\mathrm{U}}_{2}$
Guangzhou	1.00	98.26	− 753.55	− 151.93	− 132.20	− 221.49	− 2517.77	− 88.21
Shenzhen	1.36	0.00	0.00	0.00	0.00	− 10,123.66	0.00	0.00
Zhuhai	0.42	− 10.93	− 1934.05	− 248.16	− 145.87	0.00	− 6644.07	− 1243.55
Zhaoqing	0.44	− 97.12	− 2415.94	− 133.59	− 2758.56	0.00	− 7894.78	− 882.49
Dongguan	0.78	− 113.60	− 383.44	− 345.99	− 70.63	0.00	− 17,347.92	− 768.63
Foshan	0.84	− 11.06	− 708.13	− 77.00	− 71.75	− 138.52	− 6931.63	− 337.00
Huizhou	0.27	− 116.10	− 3373.49	− 1053.72	− 1199.59	0.00	− 14,598.93	− 1142.12
Jiangmen	0.27	− 123.74	− 2369.38	− 573.22	− 1891.14	0.00	− 18,285.91	− 1169.36
Zhongshan	0.40	− 60.26	− 1830.49	− 415.47	− 127.47	0.00	− 15,228.82	− 521.97
2005	0.75	− 41.41	− 41.75	− 165.81	− 441.28	− 476.47	− 3786.60	− 167.78
2006	0.72	− 41.96	− 72.81	− 232.24	− 478.63	− 566.74	− 5895.78	− 206.73
2007	0.63	− 56.32	− 225.51	− 252.65	− 751.35	− 632.52	− 10,271.01	− 365.38
2008	0.62	− 49.65	− 363.14	− 217.96	− 755.49	− 696.11	− 10,368.54	− 477.91
2009	0.71	− 61.62	− 380.19	− 329.43	− 399.84	− 904.94	− 4663.33	− 507.37
2010	0.63	− 81.63	− 629.43	− 341.17	− 767.81	− 1071.95	− 7252.87	− 540.07
2011	0.61	− 68.43	− 903.28	− 394.95	− 773.34	− 1180.65	− 9444.75	− 957.10
2012	0.60	− 62.89	− 1220.28	− 428.53	− 781.96	− 1232.09	− 11,370.01	− 725.43
2013	0.55	− 53.82	− 2060.76	− 290.63	− 798.47	− 1406.52	− 15,277.06	− 942.17
2014	0.54	− 53.42	− 2547.22	− 312.41	− 801.91	− 1580.96	− 18,875.71	− 1009.79
2015	0.67	− 19.35	− 2043.95	− 303.89	− 765.28	− 1794.13	− 8055.33	− 635.03
2016	0.66	− 22.39	− 2406.68	− 321.33	− 769.26	− 1915.90	− 9784.01	− 741.82
2017	0.64	− 29.42	− 2793.25	− 369.54	− 719.95	− 1252.62	− 10,441.88	− 935.58
2018	0.64	− 31.60	− 3111.89	− 381.57	− 719.63	− 1343.64	− 11,501.60	− 1107.44
2019	0.64	− 50.31	− 4147.36	− 656.34	− 937.81	− 1417.53	− 12,094.56	− 935.94
Overall	0.64	− 48.28	− 1529.83	− 333.23	− 710.80	− 1164.85	− 9938.87	− 683.70

From the temporal perspective, the average energy efficiency of the Pearl River Delta has shown a downward trend due to the increasing input redundancy and excessive unexpected output value since 2005. As for the growth trends of energy efficiencies from 2005 to 2019 of the nine cities, the regional differences are obvious from Fig. 1. The overall trends of Zhuhai, Jiangmen, Huizhou, and Zhongshan are nearly constant, with less fluctuation around the average values, while Zhuhai had a sudden increase in 2019. By analyzing the slack variables of these four cities from Table 3, this paper deduces that the main reasons for their decline are the scarce changes of the redundancy of capital stock and energy consumption and the increase of excessive emissions of two unexpected outputs. Besides, the overall trends of Guangzhou and Shenzhen are stable, with slight upward trends, while there has been an obvious sudden drop in recent 2 years for both of them caused by excessive input and unexpected output. Furthermore, compared with other cities, the energy efficiency of Zhaoqing, Dongguan, and Foshan fluctuated greatly from 2005 to 2019, showing extreme instability.

From the regional perspective, the average energy efficiency of the whole Pearl River Delta from 2005 to 2019 is less than 1, with an average of 0.6405, which indicates inefficiency energy using of the Pearl River Delta. The calculation results in Table 2 shows that Shenzhen and Guangzhou in the Pearl River Delta region are both energy efficient cities, and Shenzhen is the most efficient one, with an average of 1.3563. From Table 3, it is obvious that Shenzhen can further improve its energy efficiency by increasing output. However, the average energy efficiencies of the other cities from 2005 to 2019 are all lower than 1, showing absolutely inefficient. Among them, Zhuhai, Jiangmen, Huizhou, and Zhongshan are all low energy efficiency cities with overall downward trends. Compared with other cities, the energy efficiency values of these four cities are the lowest, and the overall energy efficiency values decrease in order with Zhongshan, Zhuhai, Jiangmen, and Huizhou. According to Table 3, their low energy efficiency is mainly due to excessive unexpected output and high input redundancy. In addition, due to the large fluctuations in the annual growth of input and unexpected output, the energy efficiency of Zhaoqing, Dongguan and Foshan fluctuated greatly from 0.3 to 1.1 for the 15 years, while their average energy efficiencies are still less than 1, which indicates inefficient.

### Calculation results of Malmquist index and the decomposition indexes

**Fig. 2:**
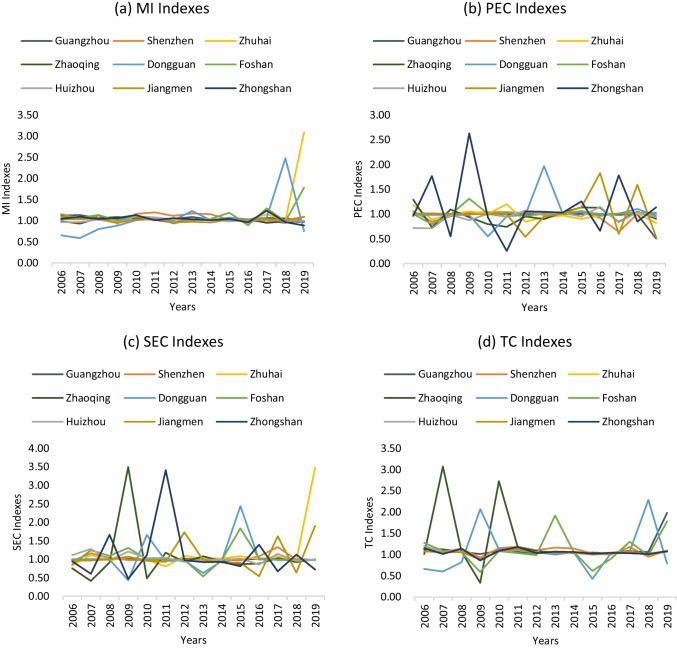
Malmquist indexes and its decomposition indexes of all regions

**Fig. 3:**
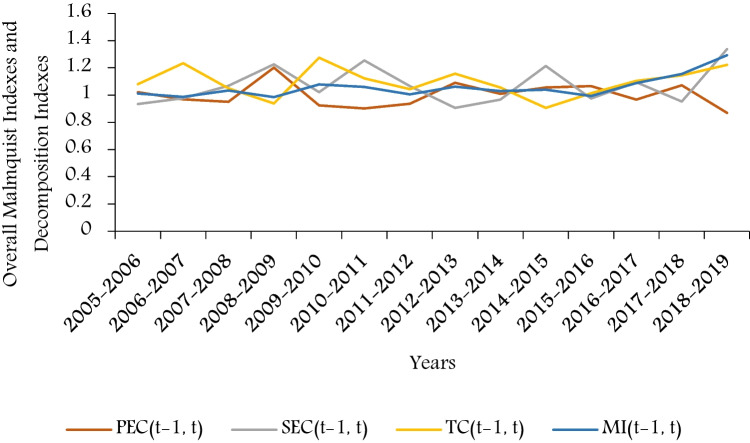
Overall Malmquist indexes and decomposition indexes

Based on the energy efficiencies calculated by the MAXDEA software, the total factor energy efficiency change in each period is estimated through the serial Malmquist indexes (MI) of each city from 2006 to 2019, and the indexes are further decomposed to calculate the technical progress indexes, pure technical efficiency indexes and scale efficiency indexes of each region in each year compared with the previous year by related calculation software. Through the calculation by MAXDEA software, each cities’ Malmquist indexes and decomposition indexes from 2006 to 2019 are shown in Fig. 2. The average values of Malmquist indexes and their decomposition indexes of each city and each period are shown in Table 4, and their overall trends and growth rates of each city are shown in Fig. 3 and Table 5. The results of super-efficiency SBM model show that whether the region is energy efficiency or not. Through the values of MI indexes, it can be illustrated that whether the energy efficiencies are progressive or retrogressive by observing whether they are greater or less than one. The results of decomposition indexes can indicates that which factor contributes the most to energy efficiency, or which factor is the main reason for inefficiency by comparing their values.

**Table 4:** Average slack movement of Malmquist indexes of each region and each period

DMU	MI(*t*-1, *t*)	PEC(*t*-1, *t*)	SEC(*t*-1, *t*)	TC(*t*-1, *t*)
Guangzhou	1.0568	0.9656	0.9996	1.1261
Shenzhen	1.0760	0.9737	1.0266	1.0887
Zhuhai	1.1801	0.9827	1.1804	1.0469
Zhaoqing	1.0015	0.9649	1.0639	1.2691
Dongguan	1.0422	1.0351	1.0694	1.0788
Foshan	1.1126	1.0122	1.0582	1.1163
Huizhou	1.0098	0.9417	1.0362	1.0506
Jiangmen	1.0127	1.0128	1.0798	1.0378
Zhongshan	1.0375	1.1346	1.1255	1.0535
2005–2006	1.0114	1.0215	0.9344	1.0803
2006–2007	0.9862	0.9695	0.9765	1.2351
2007–2008	1.0329	0.9497	1.0688	1.0502
2008–2009	0.9854	1.2026	1.2253	0.9378
2009–2010	1.0783	0.9238	1.0220	1.2739
2010–2011	1.0599	0.9025	1.2536	1.1220
2011–2012	1.0062	0.9360	1.0665	1.0447
2012–2013	1.0629	1.0900	0.9053	1.1577
2013–2014	1.0303	1.0092	0.9670	1.0559
2014–2015	1.0390	1.0562	1.2139	0.9064
2015–2016	0.9937	1.0667	0.9751	1.0144
2016–2017	1.0891	0.9661	1.0954	1.1045
2017–2018	1.1558	1.0729	0.9530	1.1448
2018–2019	1.2920	0.8696	1.3381	1.2225
Overall	1.0588	1.0026	1.0711	1.0964

**Table 5:** Malmquist indexes’ growth rate of each city over the 15 years (%)

DMU	MI(*t*-1, *t*)	PEC(*t*-1, *t*)	SEC(*t*-1, *t*)	TC(*t*-1, *t*)
Guangzhou	− 0.89	− 3.85	− 0.07	6.31
Shenzhen	0.16	1.31	1.05	0.49
Zhuhai	15.85	− 1.61	21.99	0.34
Zhaoqing	0.03	0.19	31.30	55.95
Dongguan	9.51	6.21	22.75	20.36
Foshan	6.82	1.22	7.94	13.09
Huizhou	0.38	3.52	0.43	− 0.54
Jiangmen	0.53	10.92	25.46	0.28
Zhongshan	− 0.61	53.77	28.62	0.24
Overall	3.53	7.97	15.50	10.72

It can be seen from Fig. 3, except from 2005 to 2006, from 2007 to 2008, and from 2015 to 2016, the overall Malmquist indexes of the Pearl River Delta region are all greater than 1, and the overall trend is upward, with a growth rate of 3.53%. This shows that although the average value of energy efficiency of the Pearl River Delta shows downward trend since 2005 due to the violent downward fluctuation of some cities for some time, the Pearl River Delta’s energy efficiency level is actually increasing for the 15 years. It can also be seen from Table 4 and Table 5 that although the average values of the technical progress index, pure technical efficiency index and scale efficiency index of the Pearl River Delta from 2006 to 2019 fluctuate around 1, their overall average value over the 15 years are greater than 1, showing an overall growth trend, of which the scale efficiency index increased the fastest.

As for the Malmquist indexes among the nine cities, it is obvious that the fluctuation amplitude of the Malmquist index’s values of all cities are slight and their values are close, while Dongguan, Zhuhai and Foshan have a sudden increase in recent 2 years. It can also be seen from Table 4 and Fig. 2 that although the Malmquist indexes of all cities have been fluctuated around 1 since 2006, the average Malmquist index of each city from 2006 to 2019 was greater than 1, indicating that the total factor energy efficiency of all cities showed an upward trend on the whole. Meanwhile, in addition to Guangzhou and Zhongshan, Malmquist indexes’ growth rates of other cities in the past 15 years are greater than 0, of which Zhuhai has the fastest growth rate of total factor energy efficiency.

However, the regional differences of Malmquist decomposition indexes among the nine cities are tremendous. From Fig. 2, it is obvious that Zhongshan, Dongguan, Jiangmen, and Foshan’s pure technical efficiency’s values have violently fluctuated around 1 since 2006, while the other cities’ are stable above 1. Except Guangzhou, Shenzhen, Zhuhai, Zhaoqing, and Huizhou, the rest cities’ average pure technical efficiency indexes are greater than 1, indicating overall improvement of pure technical efficiency. As for the scale efficiency indexes, expect Guangzhou, Huizhou, and Shenzhen, the rest cities also fluctuated around 1 for the 15 years, showing high instability. The average scale efficiency indexes of all cities except Guangzhou are greater than 1, showing overall progress of scale efficiency. Besides, except Zhaoqing, Dongguan and Foshan, the overall trends of the rest cities’ technological progress show stable, indicating the stable contribution levels to the improvement of energy efficiency. The average values of technological progress indexes for all cities are greater than 1, showing their high technical progress growth rate.

As for the main contribution indexes to the improvement of energy efficiency, from Table 4 and Table 5, it can be seen that the average contribution of 5.68% improvement in Guangzhou’s total factor energy efficiency comes from the change in technological progress, as only its technological progress are greater than 1 and have an overall upward trend from 2006. Its average technological progress index value from 2006 to 2019 is 1.1261, and the growth rate is 6.31%, while Guangzhou’s pure technical efficiency and scale efficiency showed a downward trend and most of them are less than 1 during this period. Besides, technological progress’ changes in Shenzhen, Zhaoqing, Dongguan, Foshan, and Huizhou are the main contribution indicators of total factor energy efficiency growth, while the overall downward trends of technological efficiency in Shenzhen, Zhaoqing, and Huizhou have inhibited their total factor energy efficiency growth. The main contribution index of total factor energy efficiency growth in Zhuhai and Jiangmen in the past 15 years is the change of scale effect, while the change index of pure technical efficiency in Zhongshan is the main contribution index of total factor energy efficiency growth.

## Discussion

In this part, according to the empirical results, this paper discusses the energy efficiency condition of each city in the Pearl River Delta.

On the whole, the overall energy efficiency of the Pearl River Delta did not reach the efficiency frontier from 2005 to 2019, indicating that the economy of the Pearl River Delta region is still dominated by the extensive growth mode of high input and low output. Furthermore, there are huge differences in energy efficiencies among cities. Among the nine cities, only Shenzhen and Guangzhou are basically at the forefront of energy efficiency, showing high efficiency of energy utilization, and the energy efficiency of Shenzhen is much higher than that of Guangzhou. Meanwhile, it is worth to note that Guangzhou has been using high-efficiency energy until 2018, while due to the redundancy of capital stock and energy consumption and the excessive discharge of unexpected outputs such as sewage, its energy efficiency fell to 0.51 in 2019. It is necessary to deal with the pollution emission in a timely manner and reduce the capital stock investment and energy consumption to prevent its further decline in energy efficiency. In addition, although the energy efficiency of Dongguan, Zhaoqing, and Foshan fluctuated between efficiency and non-efficiency in the past 15 years with huge range, their growth rates of overall energy efficiency were 2.74%, 6.89%, and 9.37% respectively, illuminating that the three cities are slowly improving their energy efficiencies. However, the problem of instability of those cities should be noticed by local government. In order to steadily improve the energy efficiency of the region, local governments need to strengthen talent introduction plans and improve local employment benefits. At the same time, they need to strengthen the monitoring of pollution emissions to prevent one-time excessive emissions and other issues. Nevertheless, the energy efficiencies of Zhuhai, Huizhou, Jiangmen, and Foshan are far from reaching the efficiency frontier, and there are a lot of invalid losses in energy use. The local government needs to pay more attention on their problems of input redundancy and unexpected excess output than the other regions.

From the Malmquist index and its decomposition results, it is obvious that the overall energy efficiency of the Pearl River Delta region is steadily rising from 2005 to 2019, showing positive progress. However, due to the great differences in industrial structure, industrial scale and energy technology among cities in the Pearl River Delta, the driving forces of total factor energy efficiency growth in different cities are also various. It is worth to mention that the growths of total factor energy efficiency in Guangzhou, Shenzhen, Zhaoqing, Foshan, and Huizhou are mainly due to the role of technological progress, while the regression of pure technical efficiency is the main factor that slows down the growth of total factor energy efficiency in Guangzhou, Shenzhen, Zhuhai, Zhaoqing, and Huizhou, and it is also the least contribution part of other cities except Zhongshan. This also highlighted that the over pure technical efficiency is obvious the weakest part. Compared with other decomposition indexes, the overall value of this index fluctuates more and is relatively small. This indicates that although the energy efficiency technology of cities in the Pearl River Delta is constantly improving, the technical level of most regions is obviously not up to the level of effective energy consumption; hence, local governments still need to specifically encourage energy technology innovation and progress. As for the scale efficiency indicators with the highest positive growth rate at 15.50% compared with other indicators, the result indicates that the market scale of the Pearl River Delta region is constantly developing and has a positive role in promoting the overall total factor energy efficiency.

## Conclusion

Using the panel data of nine cities in the Pearl River Delta from 2005 to 2019, this paper analyzes the changes and causes of energy efficiency and total factor energy efficiency in the Pearl River Delta cities through super-efficiency SBM model and Malmquist index method, and draws the following conclusions.

In conclusion, firstly, the Pearl River delta needs to further strengthen energy conservation and emission reduction policies, especially for the lowest energy efficiency areas including Zhuhai, Jiangmen, Huizhou, and Zhongshan. The government needs to further tighten emission indicators such as sewage discharge and industrial exhaust discharge, and encourage the reduction of fossil energy consumption. Secondly, as inefficient energy efficiency technology has invariably been the difficulty that all cities in the Pearl River Delta need to overcome, government still need to continue to vigorously encourage energy technology innovation, optimize the industrial structure and energy structure, encourage the use of clean energy, and support technologies that reduce the use cost of clean energy. Finally, due to the great differences in industrial structure and economic development among cities in the Pearl River Delta, it is necessary to adjust them based on local industrial structure and economic situation of each city to avoid the situation that one-size-fits-all situation when considering energy conservation and emission reduction indicators.

## Further research

Firstly, further research will collect data in depth. Due to the serious lack of data in Chinese cities, the research period of this paper can only be taken from 2005 to 2019. Further research will try to complete the data by some data completion methods, or measure or replace incomplete data by other data. Secondly, further research will further deepen the selection of variables. Due to the problem of data availability, this paper cannot include all the unexpected output measurement indicators affecting energy efficiency in the model, such as solid pollutants and dust emissions. Finally, further research will expand the scope of research. The purpose of studying energy efficiency is to optimize the local industrial chain and improve the environment. Further research will explore energy efficiency issues in other regions of China, so as to encourage coordinated development of green economy in all regions of China.

## Data Availability

The datasets used and analyzed during the whole study are available from the public website.
